# Advantages of a multi-state approach in surgical research: how intermediate events and risk factor profile affect the prognosis of a patient with locally advanced rectal cancer

**DOI:** 10.1186/s12874-018-0476-z

**Published:** 2018-02-13

**Authors:** G. Manzini, T. J. Ettrich, M. Kremer, M. Kornmann, D. Henne-Bruns, D. A. Eikema, P. Schlattmann, L. C. de Wreede

**Affiliations:** 1 Department of General and Visceral Surgery, University Hospital of Ulm, Albert-Einstein-Allee 23, 89073 Ulm, Germany; 2 Department of Internal Medicine, University Hospital of Ulm, Ulm, Germany; 3 Department of Medical Statistics and Bioinformatics, Leiden University Medical Center (LUMC), Leiden, Netherlands; 4 Department of Medical Statistics, Informatics and Documentation, University of Jena, Jena, Germany

**Keywords:** Multi-state model (msm), Dynamic prediction, Rectal cancer (RC), Local recurrence (LR), Distant metastasis (DM)

## Abstract

**Background:**

Standard survival analysis fails to give insight into what happens to a patient after a first outcome event (like first relapse of a disease). Multi-state models are a useful tool for analyzing survival data when different treatments and results (intermediate events) can occur. Aim of this study was to implement a multi-state model on data of patients with rectal cancer to illustrate the advantages of multi-state analysis in comparison to standard survival analysis.

**Methods:**

We re-analyzed data from the RCT FOGT-2 study by using a multi-state model. Based on the results we defined a high and low risk reference patient. Using dynamic prediction, we estimated how the survival probability changes as more information about the clinical history of the patient becomes available.

**Results:**

A patient with stage UICC IIIc (vs UICC II) has a higher risk to develop distant metastasis (DM) or both DM and local recurrence (LR) if he/she discontinues chemotherapy within 6 months or between 6 and 12 months, as well as after the completion of 12 months CTx with HR 3.55 (*p* = 0.026), 5.33 (*p* = 0.001) and 3.37 (*p* < 0.001), respectively. He/she also has a higher risk to die after the development of DM (HR 1.72, *p* = 0.023). Anterior resection vs. abdominoperineal amputation means 63% risk reduction to develop DM or both DM and LR (HR 0.37, *p* = 0.003) after discontinuation of chemotherapy between 6 and 12 months. After development of LR, a woman has a 4.62 times higher risk to die (*p* = 0.006). A high risk reference patient has an estimated 43% 5-year survival probability at start of CTx, whereas for a low risk patient this is 79%. After the development of DM 1 year later, the high risk patient has an estimated 5-year survival probability of 11% and the low risk patient one of 21%.

**Conclusions:**

Multi-state models help to gain additional insight into the complex events after start of treatment. Dynamic prediction shows how survival probabilities change by progression of the clinical history.

**Electronic supplementary material:**

The online version of this article (10.1186/s12874-018-0476-z) contains supplementary material, which is available to authorized users.

## Background

The medical history of a cancer patient is very complex and often includes different intermediate events, as for example the development of distant metastasis (DM) or local recurrence (LR) after surgery of the primary tumor, which plays a central role in influencing the survival prognosis. Standard survival analysis can analyze separately different endpoints but fails to give insight into what happens to a patient after a first event [1]. Multi-state models are an extension of classical survival analysis which allows adjustment to the prediction of survival duration of the patient in the course of time by incorporating new information regarding the progression of the medical history and to better understand how prognostic factors influence the different phases of the disease/recovery process [2]. A multi-state model (msm) is a model for time-to-event data in which all individuals start at one or possibly more starting states and eventually may end up in one (or more) absorbing or final state(s). In between, intermediate states can be visited, possibly more than once. Some individuals are censored before they reach an absorbing state [2, 3]. For example, the starting state could represent the time of discovery of a disease, the final state usually is death, intermediate states reflect all relevant treatments or disease stages or generally clinical events between starting state and final state. These models are very helpful in clinical decision making because they allow the prognosis of the patient to be updated according to the progression of the disease.

The course of the events between diagnosis of cancer, the many possible intermediate events like a surgical intervention, the development of metastasis, the commencement of chemotherapy (CTx) and, in the worst case, the death of a patient as final event, can be described very well by a multi-state model. This relatively new method for analysis of survival data is still not well known in the medical world. The aim of this study was to implement a multi-state model on data of patients with rectal cancer to illustrate the advantages of multi-state analysis in comparison to standard survival analysis.

The prognosis of rectal cancer has been improved significantly over the past decades as reported by De Angelis et al.; in 2014 with an increase in the 5-year overall survival from 1999 to 2001 of 52.1% (51.6–52.6) to 2005–07 of 57.6% (57.1–58.1), respectively [4]. This can be explained by the improvement of surgical techniques with the introduction of total mesorectal excision (TME) [5–11] and of MRI as diagnostic instrument [12] as well as multidisciplinary treatment with better neoadjuvant radiochemotherapy and adjuvant CTx [13]. Regarding locally advanced rectal cancer (stage II and III) this multidisciplinary work increased the 5-year cause-specific survival rates from 53.4% for patients treated between 1981 and 1986 to 89.8% for patients treated between 2007 and 2011 [13].

As the clinical history of rectal cancer patients can be well described with a msm, we analyzed the outcomes of patients from the multicentric FOGT-2 trial [14] using a msm in order to assess the influence of certain prognostic factors like age, gender, body mass index (BMI), UICC tumor stage, tumor grade and type of operation on different phases of the disease/recovery process of the patient and to obtain more accurate predictions of long-term survival than with a standard survival model by adjusting the initial prediction in the course of time by incorporating new information regarding intermediate events [2] like completion and duration of the adjuvant CTx or development of DM. So far, clinical applications of multi-state models have been limited because of the difficulties of the analysis [2]. Putter et al. published in 2006 the first re-analysis of Breast cancer data by using a multi-state model, clearly showing the added value of this kind of analysis [1]. We present the first application of a multi-state model to data from patients with locally advanced rectal cancer with the aim to introduce the advantages of this type of analysis in the research field of abdominal surgery.

## Methods

### Aim of the study

The aim of this study was to implement the multi-state model on data of patients with rectal cancer in order to illustrate the advantage of multi-state analysis in comparison to standard survival analysis.

### Included patients

We re-analyzed data from the multicentric RCT FOGT-2 [14] by using a msm. The FOGT-2 trial was set up to optimize adjuvant CTx of locally advanced RC (UICC stage II-pT3/T4 pN0 M0 or III-pT1–4 pNpos M0). A total of 796 patients were randomly assigned after primary surgery to three treatment arms: 5-FU alone, 5-FU + folinic acid (FA), and 5-FU + interferon-alpha (INF-α), arm A, B, and C, respectively. The complete postoperative adjuvant CTx treatment, irrespective of the randomization arm to which the patient was assigned, lasted 12 months. The following baseline covariates were considered: age, gender, BMI, UICC tumor stage, tumor grade and operation type (abdominoperineal amputation vs. anterior resection). In our msm study *n* = 471 patients (59.2%) were included for whom duration of the adjuvant CTx was documented and the set of all baseline covariates was complete (Additional file 1: Appendix A).

#### Ethics

For the present analysis the original data set of the FOGT-2 trial was used. This set is stored in anonymous form at the Clinic of General and Visceral Surgery and is readily available for access as Excel table. The original study (FOGT-2) conformed to the International Conference on Harmonisation/WHO Good clinical practice and was approved by the Ethics Committee of the University of Ulm (#87/91). All patients gave informed consent to participate, were informed and agreed to subsequent anonymous data analysis. Therefore no additional ethics approval was required for the present evaluation.

### Statistical analysis

Baseline characteristics were summarized as median [range] for continuous variables and frequencies and percentages for categorical variables, respectively. For the whole analysis, time was measured since date of commencement of adjuvant CTx. All outcomes were estimated by means of a multi-state model (see Appendix for a general explanation of the multi-state methodology). The quantities of interest in the multi-state model were estimated in R, version 3.3.0 with library *ˋ*mstate*´* (http://www.r-project.org) [2, 15].

### The multi-state model

We designed a multi-state model composed of 8 states (Additional file 1: Appendix A, Figure A1), i.e. state 1: Event-free and alive after having received CTx for less than 6 months (starting state), state 2: Event-free and alive after at least 6 months CTx but less than 12 months (intermediate state), state 3: Event-free and alive after completion of the 12 months of CTx program (intermediate state), state 4: Early discontinuation (discontinuation of CTx before 6 months), state 5: Late discontinuation (discontinuation of CTx between 6 and 12 months), state 6: LR only and alive (intermediate state), state 7: DM only or both DM and LR and alive (intermediate state) and state 8: Death. Death is the absorbing state, implying no paths go out from that state. Each of the possible transitions from one state to another is indicated by an arrow.

The transitions between state 1 and 2, and between state 2 and 3 are of a special nature since their timing is not random, but deterministic: all patients still in the state at 6 and 12 months, respectively, make the transition at that moment. The reason for nevertheless creating these three different states is that it allows to estimate different covariate effects for the transitions originating from state 1 to 3, thus offering an attractive way to deal with time-dependent covariate effects. For a similar reason, two separate discontinuation states have been created.

For discontinuation of CTx, only the period was available (0–6 weeks, 6 weeks–3 months, 3–6 months, 6–9 months, 9–12 months). Midpoint imputation was applied for these transitions. Patients who discontinued CTx because of LR or DM made a direct transition to states 6 and 7, to avoid creating a spurious relationship between discontinuation and adverse events.

The impact of age, BMI, gender, UICC stage, tumor grade, therapy randomization arm and type of operation was assessed by means of multi-variable Cox proportional hazards regression models for all transitions separately. Covariate impact was neither modelled for the 11 transitions with 20 events or less both because not enough information was available and because of the lower relevance of these rare transitions, nor for the two deterministic transitions. The other 8 transitions had a median number of 54 observed events (range 21–150). A two-sided *p*-value < 0.05 was considered statistically significant. However, because of the large number of tests involved and the relatively limited number of events for some transitions, all tests should be considered as exploratory.

### Dynamic prediction

We defined a low and high risk patient with different constellations of risk factors and predicted their outcomes from commencement of CTx based on the results of the multi-state analysis. We also obtained dynamic (updated) predictions of survival probabilities at a fixed point in time after surgery for patients with the same sets of covariates and a given set of post-surgery events as described by Putter et al., 2007 [3].

## Results

### The multi-state model

A total of *n* = 471 patients (59.2% of the original dataset) were included in the multi-state analysis (Additional file 1: Appendix A, Figure A2, A3). Clinical and pathological characteristics are summarized in Table 1. In a total of 215 patients (45.6%), tumor recurrence was observed during follow-up: 157 patients (73%) developed DM, 33 (15.4%) LR and 25 (11.6%) both LR and DM. The number of patients does not equal the number of metastases because one patient could have metastases in different locations. In particular of the 215 patients who were diagnosed with a recurrence, 145 had a single tumor localization, 55 two, 12 three and 3 patients had four tumor localizations (Additional file 1: Appendix B, Table B1).

**Table 1:** Summary of patients’ clinical and pathological characteristics

Variable	Median [range]	*n* (%)
Age	62 year [29–85]	
BMI	25.4 kg/m^2^ [15–49.2]	
Gender		
Male		308 (65.4)
Female		163 (34.6)
Adjuvant therapy		
5-FU alone		166 (35.2)
5-FU + FA		175 (37.2)
5-FU + IFN-α		130 (27.6)
UICC tumor stage		
II		160 (34.0)
IIIa		48 (10.2)
IIIb		134 (28.5)
IIIc		129 (27.4)
Tumor grade		
1 + 2		349 (74.1)
3 + 4		122 (25.9)
Operation type		
Abdominoperineal amputation		163 (34.6)
Anterior resection		308 (65.4)

The table describes characteristics of the subgroup of patients included in the multi-state analysis (n = 471)

**Fig. 1:**
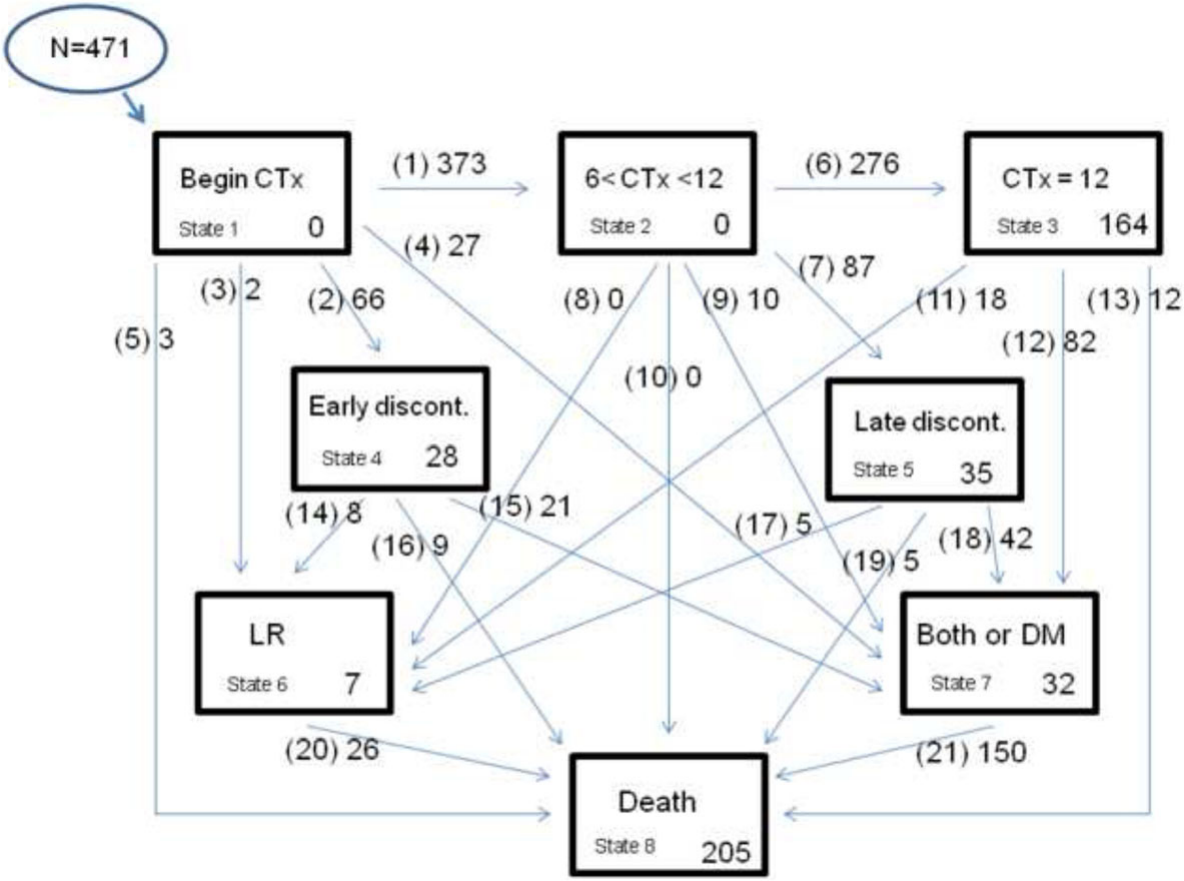
Graphical representation of the multi-state model. A total of 471 patients entered the model. A total of eight states are included: event-free and alive after beginning the CTx, alive after at least 6 months CTx but less than 12, event-free and alive after the completion of the 12 months CTx schema, discontinuation of CTx before 6 months without further events, discontinuation of CTx between 6 and 12 months and no further events, LR only and alive, DM only or LR and DM and alive, death. The number within each state indicates the number of patients in that state at the end of follow-up. For example at the moment of censoring 0 patients were in state 1 and 2 and 32 in state 7. Arrows indicate the transitions from one state to another. Numbers in () next to the arrows indicate the transition number. Numbers next to the arrows indicate the number of patients experiencing each transition. For examples 276 patients move from state 2 to state 3 and 150 patients move from state 7 to state 8. LC: local recurrence. DM: distant metastasis

Figure 1 shows the distribution of the patients at the end of the follow-up in the msm. A total of 266 patients (56.5%) were censored. Death of 205 patients (43.5%) was observed during the follow-up time. 276 (58.6%) patients completed the 12 months CTx. Reasons for discontinuation of CTx for the remaining 195 patients (41.4%) were: death within 12 months from the beginning of CTx in absence of other events (4 patients), development of LR, DM or both (74 patients), toxicity (23 patients), request of the patient (68 patients), a combination of side effects and request of patient (7 patients), the development of a second tumor other than colorectal (2 patients), other reasons (8 patients), unknown reason (9 patients).

The regression models for the transition hazards yielded the following results, which are entirely reported in Table 2. A patient with tumor stage UICC IIIc has a higher risk to develop distant metastasis (DM) or both DM and local recurrence (LR) (state 7) than a patient in stage UICC II if he/she discontinues chemotherapy within 6 months (state 4) or between 6 and 12 months (state 5), as well as after the completion of 12 months CTx (state 3) with HR 3.55 (95%-Confidence Interval (CI) [1.16; 10.86], *p* = 0.026), 5.33 (95%-CI [2; 14.19], *p* = 0.001) and 3.37 (95%-CI [1.82; 6.23], *p* < 0.001), respectively. This patient also has a higher risk to die after the development of DM or both DM and LR (HR 1.72, 95%-CI [1.08; 2.75], *p* = 0.023).

**Table 2:** Hazard Ratios for transitions in the multi-state model

Transition		From begin of CTx to ED		From begin of CTx to both LR and DM or DM only		From 6 < CTx < 12 to LD		From CTx = 12 to both LR and DM or DM only	
**A**									
Risk factor	Categories	*HR (95%CI)*	*p*	*HR (95%CI)*	*p*	*HR (95%CI)*	*p*	*HR (95%CI)*	*p*
Age (per 10 yrs)		0.91 (0.69; 1.2)	0.512	1.06 (0.69; 1.62)	0.794	1.05 (0.81; 1.36)	0.709	1.03 (0.79; 1.34)	0.821
Gender	male	1		1		1		1	
	female	1.47 (0.9; 2.4)	0.125	1.72 (0.8; 3.71)	0.167	0.69 (0.43; 1.13)	0.142	0.81 (0.5; 1.31)	0.396
Grading	1 + 2	1		1		1		1	
	3 + 4	0.78 (0.42; 1.45)	0.431	2.03 (0.9; 4.58)	0.089	1.28 (0.78; 2.08)	0.331	0.86 (0.5; 1.48)	0.59
UICC Stage	II	1		1		1		1	
	IIIa	1.42 (0.65; 3.1)	0.381	0.49 (0.06; 4.16)	0.516	0.74 (0.3; 1.79)	0.499	1.31 (0.55; 3.15)	0.544
	IIIb	1.06 (0.57; 1.98)	0.846	1.05 (0.33; 3.3)	0.94	1.14 (0.66; 1.98)	0.643	2.38 (1.31; 4.35)	**0.005** ^*^
	IIIc	1.01 (0.52; 1.98)	0.969	2.16 (0.78; 6.03)	0.14	1.56 (0.9; 2.7)	0.116	3.37 (1.82; 6.23)	**< 0.001** ^*^
BMI (per kg/m^2^)		0.99 (0.93; 1.06)	0.831	0.92 (0.83; 1.02)	0.116	0.98 (0.92; 1.04)	0.467	1 (0.94; 1.06)	0.982
Adjuvant CTx	5-FU	1		1		1		1	
	5-FU + FA	1.01 (0.54; 1.88)	0.982	0.72 (0.31; 1.69)	0.452	1.12 (0.68; 1.86)	0.661	0.92 (0.55; 1.53)	0.747
	5FU + IFN- α	1.66 (0.92; 3.02)	0.094	0.6 (0.21; 1.71)	0.34	1.38 (0.8; 2.4)	0.25	0.96 (0.54; 1.72)	0.886
Operation type	Abd.	1		1		1		1	
	amp.Anterior res.	0.81 (0.49; 1.33)	0.4	0.72 (0.33; 1.6)	0.424	1.04 (0.66; 1.62)	0.874	0.79 (0.5; 1.24)	0.307
**B**									
Transition		From ED to both LR and DM or DM only		From LD to both LR and DM or DM only		From LR to death		From both LR and DM or DM only to death	
Risk factor	Categories	*HR (95%CI)*	*p*	*HR (95%CI)*	*p*	*HR (95%CI)*	*p*	*HR (95%CI)*	*p*
Age (per 10 yrs)		1.01 (0.66; 1.53)	0.977	0.86 (0.58; 1.28)	0.462	1.85 (0.91; 3.76)	0.089	1.16 (0.96; 1.4)	0.125
Gender	male	1		1		1		1	
	female	0.88 (0.34; 2.3)	0.794	0.95 (0.42; 2.14)	0.894	4.62 (1.54; 13.89)	**0.006** ^*^	1.2 (0.83; 1.73)	0.325
Grading	1 + 2	1		1		1		1	
	3 + 4	0.9 (0.32; 2.59)	0.85	1.28 (0.64; 2.56)	0.478	1.43 (0.32; 6.38)	0.636	1.07 (0.73; 1.55)	0.737
UICC Stage	II	1		1		1		1	
	IIIa	0.63 (0.07; 5.34)	0.672	1.12 (0.21; 5.86)	0.891	0.91 (0.19; 4.44)	0.907	1.09 (0.52; 2.28)	0.815
	IIIb	1.37 (0.4; 4.66)	0.616	2.65 (0.97; 7.25)	0.059	0.76 (0.18; 3.22)	0.705	1.18 (0.72; 1.93)	0.516
	IIIc	3.55 (1.16; 10.86)	**0.026** ^*^	5.33 (2; 14.19)	**0.001** ^*^	2.82 (0.54; 14.86)	0.221	1.72 (1.08; 2.75)	**0.023** ^*^
BMI (per kg/m^2^)		0.94 (0.83; 1.07)	0.356	0.94 (0.84; 1.06)	0.306	0.94 (0.83; 1.05)	0.265	0.97 (0.93; 1.02)	0.208
Adjuvant CTx	5-FU	1		1		1		1	
	5-FU + FA	2.4 (0.78; 7.33)	0.126	0.53 (0.25; 1.13)	0.102	0.91 (0.23; 3.5)	0.885	1.29 (0.88; 1.88)	0.186
	5FU + IFN-α	0.66 (0.19; 2.36)	0.524	0.82 (0.35; 1.91)	0.639	0.69 (0.25; 1.96)	0.491	1.28 (0.82; 2)	0.284
Operation type	Abd. amp.	1		1		1		1	
	Anterior res.	0.67 (0.25; 1.77)	0.417	0.37 (0.19; 0.72)	**0.003** ^*^	0.74 (0.23; 2.41)	0.618	0.85 (0.6; 1.21)	0.37

^*^Result statistically significant at a 2-sided *p*-value < 0.05

Hazard Ratio, 95% Confidence Interval and *p*-value for each covariate and each transition with more than 20 events (*ED* Early discontinuation, *LD* Late discontinuation, *CTx* chemotherapy, *LR* local recurrence, *DM* distant metastasis, *CI* confidence interval)

Patients who underwent anterior resection have a 63% risk reduction to develop DM or both DM and LR in comparison to patients who underwent an abdominoperineal amputation (HR 0.37, 95%-CI [0.19; 0.72], *p* = 0.003) after late discontinuation. After development of LR (state 6) a woman has a 4.62 higher risk to die (state 8) in comparison to a man (HR 4.62, 95%-CI [1.538;13.890], *p* = 0.006). Similar effects of gender could not be observed for other transitions in our model. In absence of statistical significance, there is a trend that the combination-CTx schemata 5-FU/FA and 5-FU/IFN-alpha seem generally to be more effective than 5-FU alone in most transitions.

### Prediction based on the msm

**Fig. 2:**
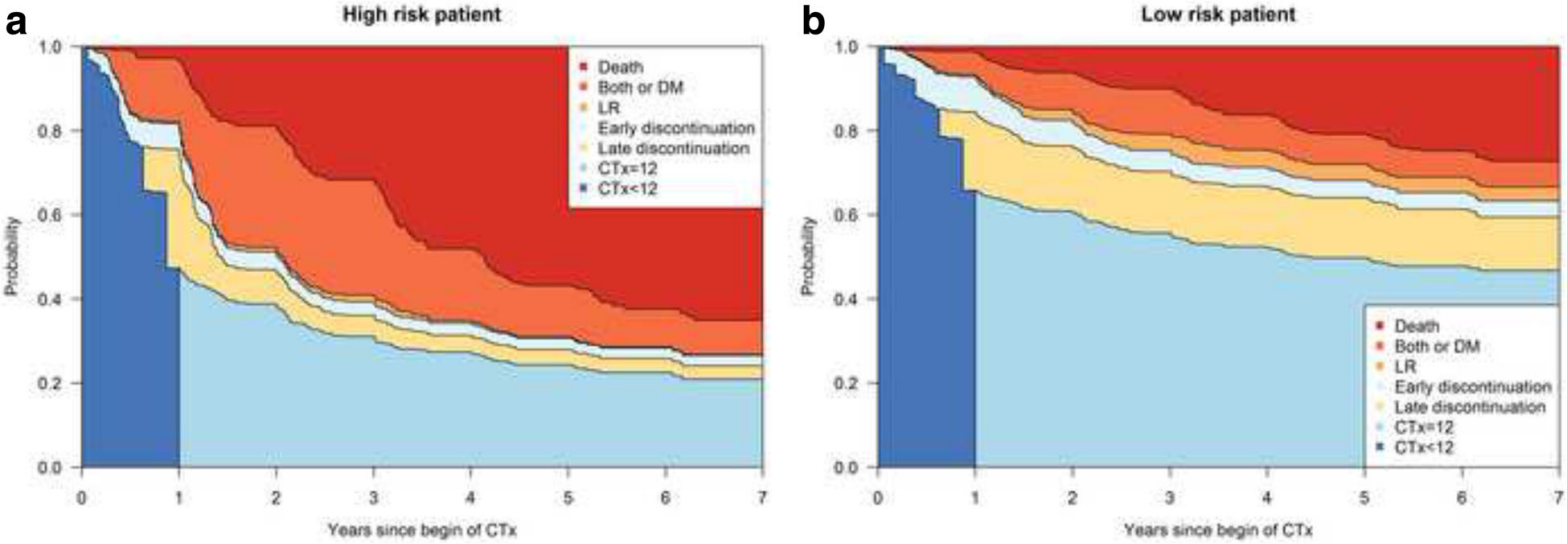
**a, b**: Stacked transition probabilities. Transition probabilities starting from state 1 - begin of CTx - at *t* = 0 for the high risk patient (**a**) and for the low risk patient (**b**) as defined in the results section. The term *´*stacked*´* means that all transition probability curves are plotted on top of each other; the difference between 2 curves indicates the transition probability from state 1 to the state with the name written between the curves. States 1 and 2 have been merged to one state ‘CTx < 12’ (meaning under treatment with CTx) since the transition between them does not represent an event with a clinical meaning

On the basis of the results of the msm, the estimated transition probabilities from commencement of CTx (when all patients are in state 1) can be calculated for reference patients. For illustrational purposes, we defined a high risk patient (tumor stage UICC IIIc, tumor grade 3–4, CTx 5-FU alone) and a low risk patient (tumor stage UICC II, tumor grade 1–2, CTx 5-FU + FA). Both patients are male, 61 years of age at the beginning of CTx, have a normal BMI of 25 and underwent anterior resection. Transition probabilities are represented in Fig. 2 a, b. Figures clearly show that the high risk patient has, for example, a higher death probability and the low risk patient has a higher probability to complete the 12 months CTx schema and to remain in that state.

### Dynamic prediction of 5-year survival probabilities

We again considered the high and low risk reference patients and we were interested in the dynamic prediction of 5-year survival probabilities. We checked how these predicted probabilities change as more information about intermediate events becomes available in the course of time. In particular we studied how 5-year survival probabilities changed when both patients stop CTx at 8 months and experience a DM 1 year after beginning the CTx and how the same probabilities changed when they complete the 12 months CTx schema and then develop DM 2 years after start of CTx.

**Fig. 3:**
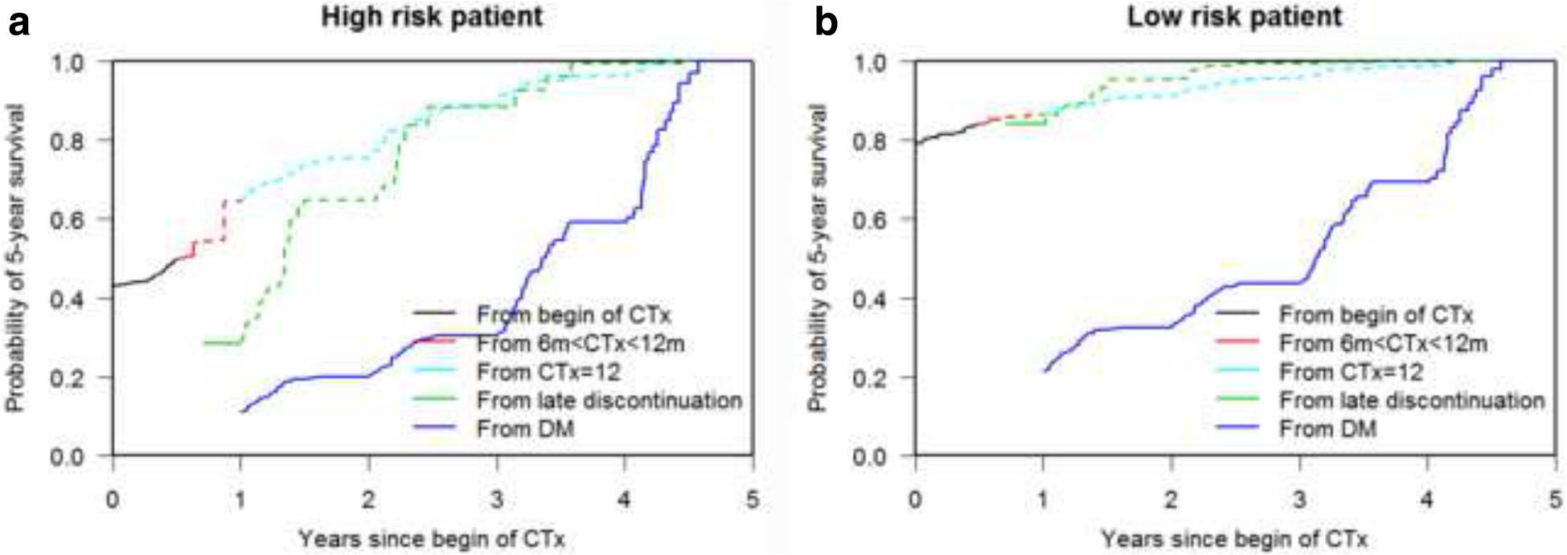
**a, b** Dynamic prediction of 5-year survival. The plots show time-dependent predictions of survival at 5 years after commencement of CTx for the high risk patient (**a**) and for the low risk one (**b**) after discontinuation of CTx at 8 months and the development of a DM at 1 year after start of CTx. Dashed curves indicate what the 5-year survival probability would have been without further intermediate events (discontinuation of CTx, DM)

**Fig. 4:**
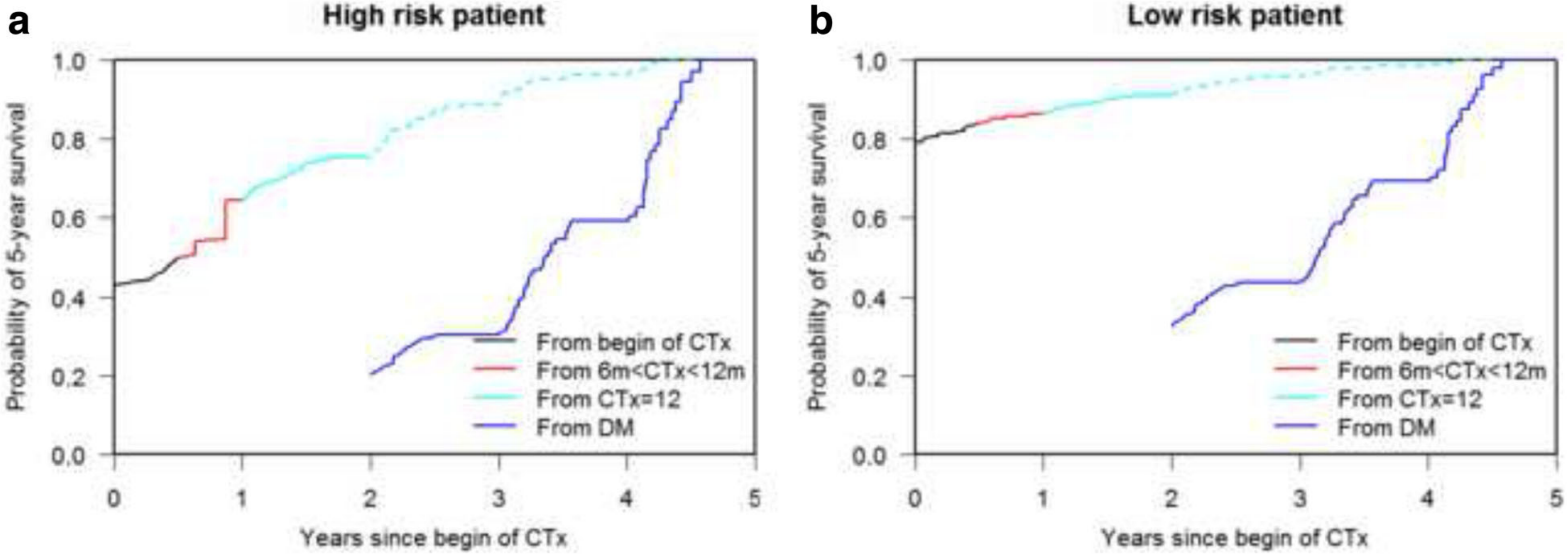
**a, b** Dynamic prediction of 5-year survival. The plots show time-dependent predictions of survival at 5 years after commencement of CTx for the high risk patient (**a**) and for the low risk one (**b**) after the completion of the 12 months CTx and the development of a DM 2 years after start of CTx. Dashed curves indicate what the 5-year survival probability would have been without DM

The plots show the 5-year survival probabilities for the high and low risk patient based on their history as described above (Figs. 3 a, b and 4 a, b). Regarding the first medical history example we can see in Fig. 3a that the high risk patient has an estimated 5-year survival probability of 43% (SE 0.07) at the beginning of CTx based only on his risk factor profile. This probability decreases dramatically to 11% (SE 0.05) at time *t* = 1 year (the moment at which he develops DM). At the same time (1 year) this probability would have been about 29% (SE 0.10) if he would not have developed DM. In Fig. 3b we show that the 5-year survival probability of the low risk patient is 79% (SE 0.04) at the beginning of CTx based only on his risk factor profile. This probability decreases to 21% (SE 0.09) when he develops DM, 10% better than the prognosis of the high risk patient. This probability would have been 84% (SE 0.05) if he would not have developed DM but had discontinued CTx at 8 months. Although the low risk patient seems to have a better survival probability with a less than 12 months lasting CTx in comparison to a 12 months lasting CTx, the curves in this study are too close to conclude that 6 m < CTx < 12 m is better than CTx = 12 months. 95%-CIs are also overlapping (Additional file 1: Appendix B, Table B2). Figures 4a and b show the survival probabilities of the two reference patients after completing the 12 months CTx schema and the development of a DM. The high risk patient (Fig. 4a) has a 5-year survival probability of 20% (SE 0.09) at the moment at which he develops DM. At the same time (2 years) this probability would have been about 76% (SE 0.08) if he would not have developed the DM but completed the CTx schema. In Fig. 4b we observe that the 5-year survival probability of the low risk patient is 33% (SE 0.13) when he develops the DM 2 years after start of CTx. By the completion of 12 months CTx in absence of a DM the survival probability from *t* = 2 years would have been 91% (SE 0.03).

## Discussion

Multi-state models are an extension of standard survival analysis, where standard survival models measure the time span from some time origin until the occurrence of the event of interest but fail to give information when the analyzed process involves more than one type of event [3].

So far most applications of multi-state models can be found in the field of hematological malignancies, in particular for the analysis of outcomes after hematopoietic stem cell transplantation, an intervention associated with many disease- and treatment-related events and therefore very suitable for multi-state modelling [16–19]. In the field of solid tumors, less applications of multi-state models can be found. Putter et al. 2006 [1] used a multi-state model to analyze survival data from women with breast cancer. Conlon et al. used a multi-state model with an incorporated cured fraction for recurrence to jointly model time to recurrence and time to death in colon cancer [20, 21]. The authors considered a total of 13,938 subjects from 12 randomized phase III adjuvant trials of locally advanced colon cancer and used a Bayesian Markov-Chain-Monte-Carlo (MCMC) technique to estimate the parameters of the multi-state cure model, like those of the Weibull model for each of the hazard rates, covariate effects for each of the hazard models and covariate effects in the logistic model for the probability of cure [20]. In a second publication, the same authors explore different ways in which the information about recurrence time and the assumptions in the multi-state model with an incorporated cured fraction for recurrence can lead to improved efficiency. Estimates of overall survival and disease-free survival can be derived directly from the model with efficiency gains obtained as compared to Kaplan-Meier estimates [21]. Other applications are described by Andersen et al. [22] who use a multi-state model for bleeding episodes and mortality in liver cirrhosis and by Mitchell et al. who show the application of a multi-state model for urinary tract infections [23].

Cancer history is maybe the best domain for the application of multi-state models in the medical field. From diagnosis, the patient can experience different events, such as surgical treatment, CTx, LR, or DM, which alter the survival probability. By better knowing the probability of having a certain event (e.g. to develop DM or LR) after the primary event (e.g. surgery for rectal cancer) in the specific case of a patient with known baseline covariates and by knowing the time at which the prediction should be done, it is possible to adapt the follow-up examinations to the risk profile of that patient.

Multi-state models offer several advantages with respect to more commonly used methods in survival analysis. The models are more flexible than Cox models with time-dependent covariates since they allow different baseline hazards for different transitions. They are more comprehensive than landmark models since all prediction timepoints are included in a single model and since sequences of events can also be analyzed. A major advantage compared to both of these methods is that the model yields estimates of probabilities of being in both intermediate and absorbing states. In our model, e.g., we can consider the relevance of predictions starting from any of the states indicating treatment discontinuation by assessing the chance that a patient with certain characteristics really is in such a state at different moments after start.

We designed a msm with eight states with the aim to investigate the effect of the covariates on the different transitions from one state to the other and the role of intermediate events in affecting the survival probability of the patients. For applying standard formulas and software to estimate the quantities of interest, it had to be assumed that the model is Markovian, i.e., that the prognosis for a patient at a certain moment depends on the state where he/she is at that moment, and not on the states previously visited or the duration in the current state. By creating two CTx states instead of one and two discontinuation states, we managed to include information about the patients’ histories (length and possible discontinuation of CTx) and assessing the differential impact of risk factors on subsequent transitions, while still keeping the attractive statistical properties of the Markov model and the possibility to fit the model with the ‘mstate’ package. It must be noted, however, that the states 6 < CTx < 12 and CTx = 12 are not, like the other states, characterized by an event that makes the patient experience a transition, but only by the passing of time; they are thus more relevant for model fitting than for describing clinical histories. Other approaches exist in which time-dependent effects of the history of the patients can be modelled without categorizing time.

A weakness of the current study is that for some of the transitions the exact transition times were not available. For discontinuation of CTx, only the period was given in the data. This event should in principle have been treated as interval-censored and be analyzed by a method suitable for this kind of data. However, to remain within the semi-parametric framework of our model, we used a pragmatic approach by applying midpoint imputation. In addition, time-to-event data regarding the development of LR, DM or both LR and DM were recorded in the original study in only 3 categories: LR, DM or both LR and DM. For the patients in this last category only the date of the second of these two events (LR or DM) was recorded. According to the current literature which states that in case of simultaneous LR and DM the prognosis is defined by the DM [24, 25], we decided to create one state named *´*DM or both LR and DM*´*. Of a total of 182 patients who entered this state, only 25 (13.7%) were categorized in the original study by the presence of both DM and LR. Time intervals between LR and DM or *viceversa* were not available for these patients, implying the exact moment when they entered the LR or DM state is unknown. Since this only affects 25 patients and since this time interval will have been short for some of them, we assume that the impact of this missing data on the estimated probabilities is limited. In addition, information about a possible second operation to treat the LR and/or the DM, which could be represented as an additional state in our model since it may have affected the prognosis of the patients, was also not recorded in the original study.

In the current study, 40% of patients had to be excluded from the multi-state analysis. Also in other contexts, the lack of detailed data describing risk factors and the course of the disease for larger groups of patients is an impediment to the development of relevant and accurate multi-state models. Therefore, a major goal of the current study is to illustrate some of the features of multi-state models to advocate their use and to improve data collection to this end. For example, the multi-state model helps to zoom in on the differential impact of the three CTx regimens on different endpoints. This information cannot be retrieved by the primary analysis of the trial.

In accordance with current literature we found that patients with higher tumor stage and/or grade have a higher risk to develop DM both after early and late discontinuation of CTx and after completion of 12 months of CTx [26]. Interesting is the result regarding the worse survival prognosis of women after the development of LR compared to that of men. However, this finding may be partly due to chance because of the low number of events in this transition (*n* = 26). For this reason we considered both the poor and good risk reference patient to be male. No specific literature exists regarding how gender affects survival after the development of LR or DM. One publication dealing with gender [27] aimed to investigate the association of gender and age with acute toxicity after radio-chemotherapy for UICC II/III rectal cancer. Although women showed higher hematologic (*p* < 0.001) and acute organ toxicity (*p* < 0.001) in comparison to men, the authors found a trend toward higher 10-year overall survival in women (62.7% vs. 58.4%, *p* = 0.066). By analyzing our data stratified for gender (by the Kaplan-Meier method), we found similar survival in women and men (log-rank test, *p* = 0.85).

Patients that underwent an abdominoperineal amputation have a higher risk to develop DM or both DM and LR. It is known and has been shown in several studies that a higher incidence of LR after abdominoperineal resection occurs compared to low anterior resection [28]. In fact, patients who need this type of surgery have a tumor which is locally advanced and most likely muscle infiltrating, which makes the accomplishment of a radical resection difficult, increasing the possibility of the development of LR due to remaining tumor tissue or cells after the operation [28]. Kornmann et al. reported in 2010 a 5-year recurrence-free survival rate for locally advanced rectal cancer of 60.4% (95% CI [55%–65.4%]) after anterior resection vs. 46.2% (95% CI [38.6%–53.5%]) after abdominoperineal amputation (*p* = 0.005) [29]. We could not demonstrate a higher risk to develop LR with this type of operation in our data. However, as previously described, we have not enough sample size in the transitions into the state LR. Finally, none of the risk factors included in the models was significantly associated with discontinuation of CTx.

Based on the results of the msm analysis and on current literature [14, 24–26, 30] we defined a high and low risk reference patient based on the baseline covariate profile, representing well the patients in the dataset.

The graphical illustrations of the 5-year predicted probabilities answer immediately the question about the relevance, in term of survival probabilities, of each event in the clinical history of the patient allowing the adjustment of the survival probabilities in the course of time. In fact, dynamic prediction allows the estimation of survival probabilities of a patient at each point in time taking into consideration all new events which take place. Additionally we showed how different combinations of covariates (high and low risk patient) define from the beginning the survival probability. Generally the prognosis got worse for all patients when they experienced DM, even if the 5-year-survival probability of survival for the low risk patient was higher than that of the high risk one, 21% vs. 11% at 1 year and 33% vs. 20% at 2 year after start of CTx, respectively.

The question about a possible correlation between duration of CTx, risk factor profile and survival probability in locally advanced rectal cancer remains open and more studies are needed. In fact, regarding the low risk patient we observed that the survival curves representing 5-year survival probability with late discontinuation vs. CTx = 12 are too close to each other to conclude that 6–12 months CTx is better than 12 months. The actual guidelines for locally advanced rectal cancer (UICC II and III) in absence of any neoadjuvant treatment, as was the case for the patients included in the FOGT-2 study, suggest a 6 months long adjuvant radio-chemotherapy with 5FU or Capecitabine [21, 22].^.^ We defined consequently in our multi-state model the three groups: CTx duration < 6 months, CTx duration between six and 12 months according to the recommendations of the S3 guidelines, CTx duration of 1 year according to the study protocol.

One promising field which is gaining more and more interest regarding response prediction to CTx is gene expression profiling [31, 32]. In a recent study published in 2015 [33], a gene expression profile was developed which was able to predict treatment response in CRC patients treated with standard CTx regimens. Indeed, patients with a favourable gene expression profile had a higher response rate (58% vs. 13%, *p* = 0.024), progression-free survival (61% vs. 13% at 1 year, HR = 0.32, *p* = 0.009) and overall survival (32 vs. 16 months, HR = 0.21, *p* = 0.003) than patients with an unfavourable predictive signature. Future studies should consider also this variable as important prognostic factor.

One partial limitation regarding the routine use of msm in surgical research is the complexity of the computer program needed for such an analysis. However very good tutorials are freely available online and explain the procedure in detail [2, 3]. Our aim was to illustrate an intuitive application of a multi-state model in the medical field with an immediate and concrete answer to the question about survival probability given a particular medical history of a patient. We think it is important to introduce multi-state models in the medical field in a way which is understandable for clinicians.

## Conclusion

In conclusion, multi-state models are a promising tool also in surgical research as they help to gain additional insight in the complex events after start of treatment and to extract more information from trial data. Risk factor profile defines from the beginning the prognosis of the patient. By using dynamic prediction, it is possible to adjust the prognosis in course of time by incorporating any type of information regarding the disease development and treatment options (intermediate events) in the clinical history of the patient. This allows the creation of treatment algorithms for each possible disease/recovery process, which can simplify the decision making process and allow a better risk-benefit calculation.

## Additional file

Additional file 1: **Appendix A.** This file contains an introduction about multi-state models as well as the graphical representation of the model we used for the analysis of the data. Additionally, a flow-chart illustrates the procedure for the selection of the patients who could be included in the analysis together with a landmark analysis to assess the likelihood of informative missing. **Appendix B** Table 1b illustrates the localization of tumor recurrence and Table B2 shows the 5-year survival probability with 95%-CI at different timepoints after start of CTx both for Late discontinuation and for CTx = 12 m for the low risk patient. (PDF 120 kb)
