# Supplementary material for: Advantages of a multi-state approach in surgical research: how intermediate events and risk factor profile affect the prognosis of a patient with locally advanced rectal cancer
Source: BMC Med Res Methodol. 2018 Feb 13;18:23. doi: 10.1186/s12874-018-0476-z (PMC5811976; doi:10.1186/s12874-018-0476-z)
Supplement: Supplementary file 1 — Appendix A. This file contains an introduction about multi-state models as well as the graphical representation of the model we used for the analysis of the data. Additionally, a flow-chart illustrates the procedure for the selection of the patients who could be included in the analysis together with a landmark analysis to assess the likelihood of informative missing. Appendix B Table 1b illustrates the localization of tumor recurrence and Table B2 shows the 5-year survival probability with 95%-CI at different timepoints after start of CTx both for Late discontinuation and for CTx = 12 m for the low risk patient. (PDF 120 kb) [file 12874_2018_476_MOESM1_ESM.pdf]

APPENDIX A

Multi-state models

A multi-state model consists of different states (indicated by boxes) and transitions (indicated by arrows). Patients experience a transition when they pass from one state to the other. Each of the possible transitions from one state to another has an associated transition hazard which is the instantaneous risk of a transition from one state (state i) to another (state j) at time t. For the analysis of the data we assumed a transition-specific Cox model with covariates fixed at baseline. This means that the model specifies different covariate effects for the different transitions, as well as separate baseline hazards for each transition. The Markov property is assumed, meaning that the hazards only depend on the present state, not on the history of the patient. The covariates do not change over time. The mathematical definition of the model is the following:

$$\alpha_{gh} (t|\mathbf{Z}) = \alpha_{gh,0}(t) \exp(\beta^T \mathbf{Z}_{gh}),$$

where *gh* indicates the transition from state *g* to state *h*, $\alpha_{gh,0}\ (t)$ is the baseline hazard for this transition, **Z** ist the vector of covariates at baseline and **Z**gh is the vector of transition-specific covariates. Regarding the time scale *t* refers to, we used a clock-forward approach to model the effect of covariates on transition g →h. This means that time *t* refers to the point in time since the patient has entered the initial state (i.e., beginning of the first cycle of CTx). The clock keeps moving forward for the patient also when intermediate events occur[3]. The transition-specific hazards are the building blocks for the transition probabilities. A transition probability is defined as $P_{gh}(s,t) = P(X(t) = h|X(s) = g)$, meaning the probability to be in state h at time t given that the patient was in state g at time s, possibly depending on covariates. In our model, for example, $P_{35}(1.5,t)$ indicates the probability of having developed a DM and not having died (state 5) by time t, given that the patient was alive and had completed the CTx schema of 12 months (state 2) at 1.5 years after start of CTx. By varying s and fixing t, it is possible to predict the future outcomes of the patient given the (possibly different) state(s) at time point *s*. In particular, based on the covariate profile we defined a high and low risk patient and compared their 5-year survival probabilities with and without discontinuation of CTx combined with the development of a DM at different moments.

Our multi-state model

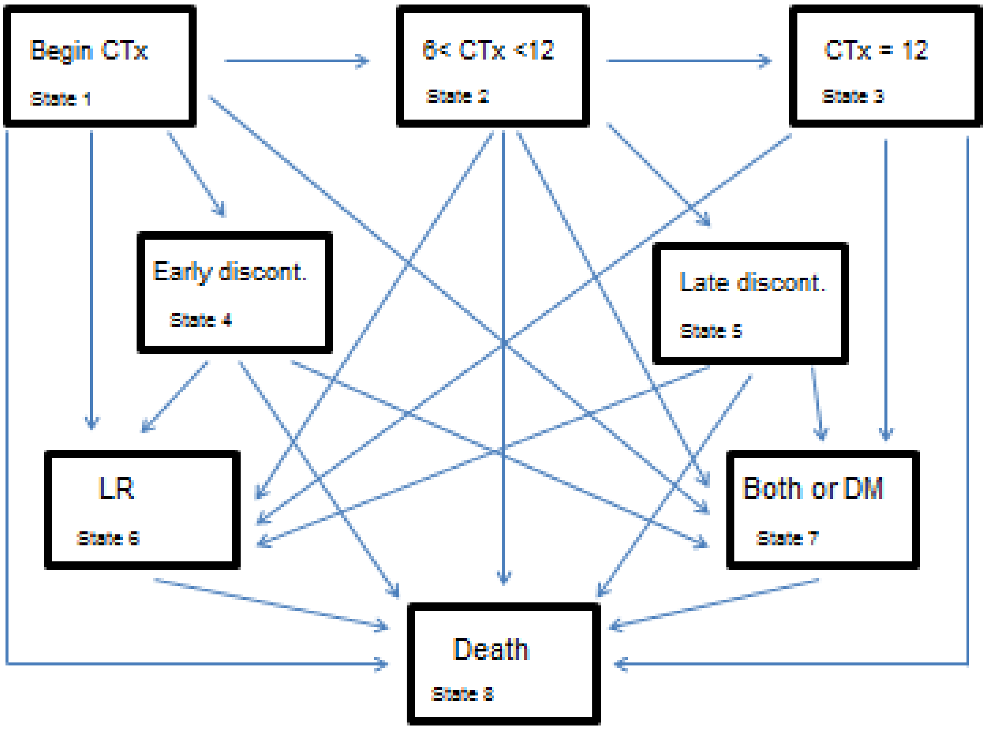

Figure A1: A graphical representation of the msm. Arrows going from one state to an other represent the transitions. LR: local recurrence. DM: distant metastasis. Both: both LR and DM. All patients start in the state ´begin CTx´. Time-to-event data regarding the development of LR, DM or both LR and DM were recorded in the original study in only 3 categories: LR, DM or both LR and DM. The 25 patients recorded in this last category could have developed first a LR and then a DM or the contrary, but only the date of the last of these two events was recorded. For this reason we have no information about possible transitions from LR or DM to both LR and DM and unfortunately we could not retrieve it retrospectively. Taking this information into consideration we decided to create one state named ´DM or both LR and DM´. A total of 182 patients entered this state, only 25 of these (13.7%) were categorized in the original study by the presence of both DM and LR. Time intervals between LR and DM or *viceversa* were not available for these patients, implying the exact moment when they entered the LR or DM state is unknown. Since this only affects 25 patients and since this time interval will have been short for some of them, we assume that the impact of these missing data on the estimated probabilities is limited.

Likeliness of informative missing

We included a total of 471 patients in our multi-state analysis as shown in the figure below.

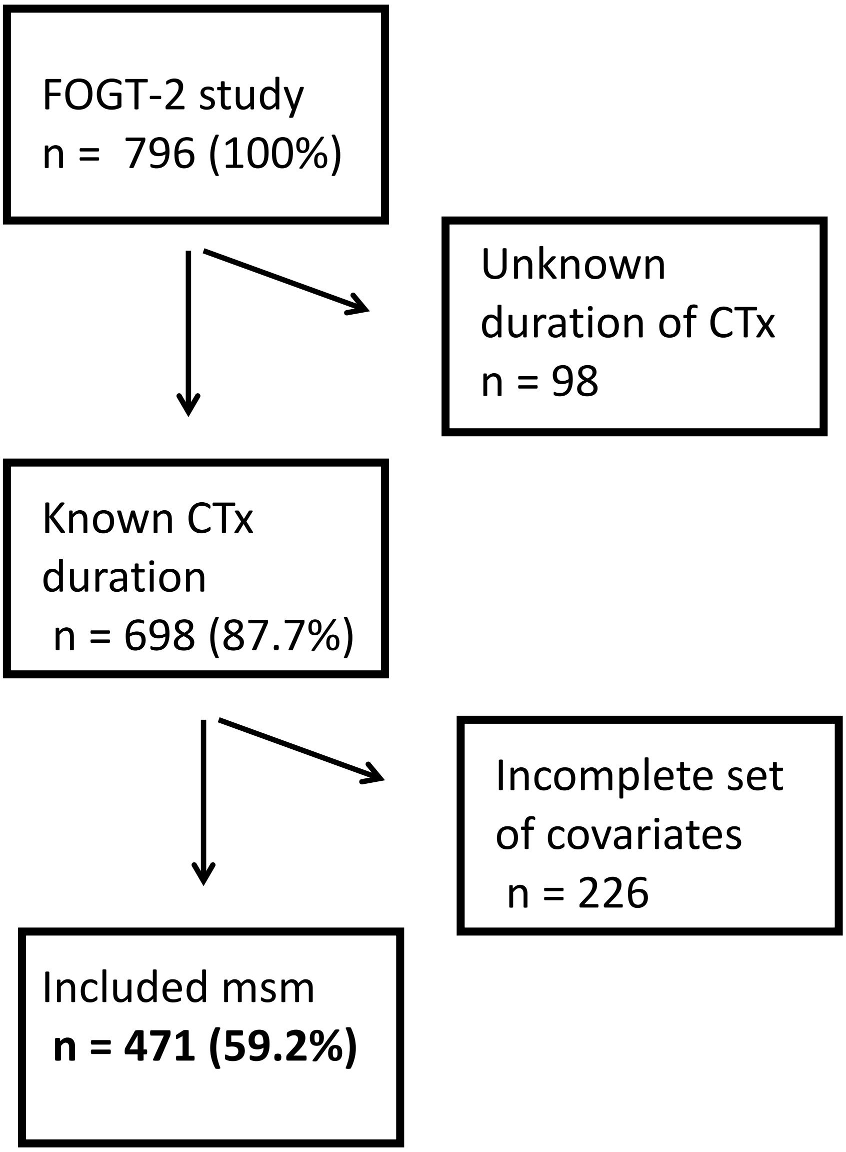

Figure A2: Patients included in the multi-state analysis

By means of a non-parametric landmark analysis at 6 months we assessed the likeliness of informative missing. Six months was chosen because before that time point no information about CTx duration could have been known. The landmark analysis from 6 months after start of therapy showed no difference in survival for the group of patients included in the multi-state model and the excluded patients (n=325) (log-rank test p-value = 0.86). There were no indications that informative missing took place. The survival curves for the two groups are shown in the **Figure** below.

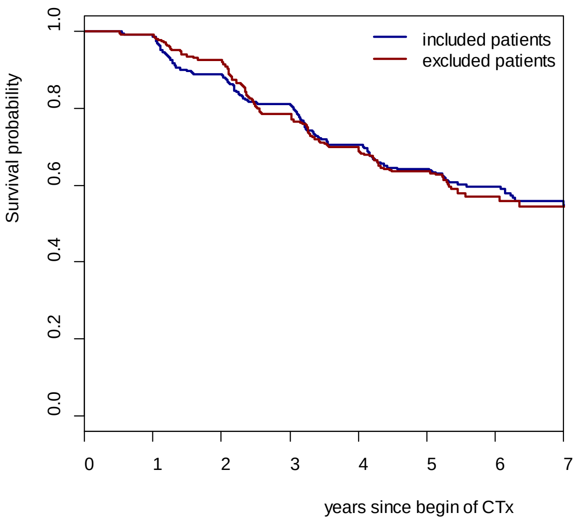

Figure A3: Landmark analysis at 6 months to assess the likelihood of informative missing. The survival curves of the patients who were included in the multi-state analysis does not differ from that of the patients excluded because of incomplete set of baseline covariates or unknown duration of CTx (p=0.86)

REFERENCES

Putter H, Fiocco M, Geskus R.B. Tutorial in biostatistics: Competing risks and multi-state models. Statist Med 2007; **26**:2389-430.

Van Houwelingen HC. Dynamic prediction by landmarking in event history analysis. Scand J Stat 2007; **34**: 70-85.

APPENDIX B

Table B1 : Localization of tumor recurrence (n=215)

	Number of patients (%)	Number of metastasis (%)
Distant metastasis	157 (73)	217 (100)
Liver		83 (38.2)
Lung		70 (32.3)
Peritoneum		12 (5.5)
Abdominal		10 (4.6)
Bones		7 (3.2)
Ascites		1 (0.5)
Other		34 (15.7)
Local recurrence	33 (15.4)	34 (100)
Pelvis		27 (79.4)
Anastomosis		7 (20.6)
Both local recurrence and distant metastasis	25 (11.6)	56 (100)
Pelvis		21 (37.5)
Liver		7 (12.5)
Peritoneum		5 (8.9)
Lung		4 (7.1)
Bones		4 (7.1)
Abdominal		4 (7.1)
Ascites		3 (5.5)
Anastomosis		1 (1.8)
Other		7 (12.5)

Table B2: 5-year survival probability with 95%-CI at s=1,2,3 and 4 year after start of CTx both for Late discontinuation and for CTx=12m for the low risk patient

s	S(s,5) from Late discontinuation	95%-CI from Late discontinuation	S(s,5) from CTx=12	95%-CI from CTx=12
1	0.842	[0.742; 0.941]	0.863	[0.785; 0.941]
2	0.952	[0.885; 1]	0.913	[0.847; 0.980]
3	0.992	[0.966; 1]	0.955	[0.913; 0.997]
4	0.999	[0.998; 1]	0.987	[0.968; 1]

s=time in years since start CTx (prediction moment), S(s,5)=5-year survival probability at time s, CI=confidence interval
